# Gut microbiota regulates serum metabolites in mice with nonalcoholic fatty liver disease via gut metabolites: mechanisms involving branched-chain amino acids and unsaturated fatty acids

**DOI:** 10.3389/fendo.2025.1606669

**Published:** 2025-07-22

**Authors:** Hao Qiu, Yuhang Wen, Yadan Luo, Shuya Lv, Jingrong Huang, Baoting Chen, Ruilin Lu, Lvqin He, Qian Yang, Jianhong Han, Xuefeng Yan, Manli He, Mingde Zhao, Xiaoxia Zou, Congwei Gu

**Affiliations:** ^1^ Laboratory Animal Center, Southwest Medical University, Luzhou, China; ^2^ Model Animal and Human Disease Research of Luzhou Key Laboratory, Southwest Medical University, Luzhou, China; ^3^ Department of Nutrition and Food Hygiene, School of Public Health, Southwest Medical University, Luzhou, China

**Keywords:** nonalcoholic fatty liver disease, serum metabolites, gut microbiota, gut metabolites, metabonomics, MetOrigin

## Abstract

**Introduction:**

In recent years, nonalcoholic fatty liver disease (NAFLD) has become the most common chronic liver disease globally. Studies indicate that the gut-liver axis plays an important role in the occurrence and development of this disease. Our previous studies showed that the gut microbiota and gut metabolites in mice with NAFLD changed significantly. However, it is unclear whether these changes influenced the disease process through serum metabolites.

**Methods:**

We conducted a non-targeted metabolome analysis on serum metabolites and systematically investigated the correlations between serum metabolites, gut microbiota, gut metabolites, and phenotypic index. Additionally, we traced the potential origins of serum metabolites and analyzed host-microbial interactions to elucidate the underlying mechanisms linking changes in serum metabolites with gut microbiota and gut metabolites.

**Results:**

The findings suggest that the imbalance of gut pathogenic microbiota, specifically Blautia and Helicobacter, and beneficial microbiota such as Allobaculum, in mice with nonalcoholic fatty liver disease may be an important cause of gut metabolic disorders. This disorder results in a reduction of unsaturated fatty acid content, particularly a decrease in Eicosapentaenoic Acid (EPA) and Docosahexaenoic Acid (DHA), and an accumulation of branched fatty acids in the serum. Consequently, there is a significant elevation in liver injury indices, potentially exacerbating the progression of nonalcoholic fatty liver disease and obesity in mice.

**Discussion:**

These results suggest that serum metabolites are influenced by gut microbiota and their metabolites. The variations in serum metabolites provide valuable insights into the relationship between gut microbiota and their metabolites in the context of nonalcoholic fatty liver disease.

## 1 Introduction

The incidence of nonalcoholic fatty liver disease (NAFLD) is increasing worldwide, and it has become the first cause of chronic liver disease in China. The epidemiological survey of NAFLD shows that the global prevalence of NAFLD has increased from 25.3% in 1990–2006 to 38.0% in 2016-2019, and about 1/4 of the population has NAFLD (1). Breakthrough research in recent years has revealed the close relationship between gut microbiota and host metabolism, indicating that maintaining healthy interaction between host and gut microbiota is very important for the overall health of host (2). More and more evidence shows that gut microbiota and its metabolites also play an important role in the occurrence and development of many metabolic diseases, including obesity, type 2 diabetes, NAFLD and cardiovascular diseases (3). These diseases are often closely related to changes in eating habits and lifestyles (4). The composition and function of gut microbiota are influenced by many environmental factors, such as age, diet, use of antibacterial drugs, psychological pressure, early and current environmental exposure, genetic factors and cohabitation, which together shape the diversity and stability of individual gut microbiota and have a far-reaching impact on the digestive health, immune system development, metabolic pathway and disease susceptibility of the host (5).

Among them, diet is the main environmental factor affecting the composition of gut microbiota community. Nutrients in the diet are converted into metabolites during the digestion of the host, which not only provide energy and growth substrates for gut microbiota, but also promote or inhibit the growth of it. In addition, gut microbiota can metabolize dietary components first, and the metabolites produced by them can be used by the host to form a complex interaction network. Different dietary patterns and intake have different regulatory effects on gut microbiota. For example, high-fat diet (HFD) can increase the proportion of Bacteroidetes and Firmicutes in the intestine, especially the abundance of some genera of bacteria, such as *Adercreutzia*, *Coprococcus*, *Dorea* and *Ruminococcus*, is significantly increased (6). These changes may be related to the accumulation of fat in the liver, and may lead to the decrease of the abundance of beneficial bacteria such as *Lactobacillus* and *Bifidobacterium*, as observed in the gut microbiota of NAFLD patients (7). In contrast, the Mediterranean diet, as a healthy diet, is rich in dietary fiber, monounsaturated fatty acids and ω-3 fatty acids, which can increase the abundance of *Bifidobacterium* and reduce the abundance of Gram-negative bacteria, thus improving the adipose tissue inflammation and intestinal barrier function of NAFLD. Therefore, different dietary patterns and nutrient intake can change the composition and function of gut microbiota, which produce key metabolites such as short-chain fatty acids, amino acid derivatives and bile acids through fermentation, and then affect the metabolic health and disease risk of the host through the gut-host axis.

Currently, it is believed that metabolites produced by the gut microbiota enter the circulatory system, thereby altering the serum metabolite profile. For example, short-chain fatty acids (SCFAs), bile acid metabolites, trimethylamine (TMA), and others can cross the intestinal epithelial barrier and directly enter the circulatory system via the portal vein. Additionally, bile influences the composition of serum bile acids through the enterohepatic circulation (8). These changes in serum metabolites subsequently affect the liver through various pathways. First, the accumulation of lipid intermediates such as free cholesterol in the liver interacts with YAP-TAZ, driving sterile inflammatory responses. This process, which exhibits lipotoxic effects, is considered a key pathological mechanism of lipotoxicity in NAFLD (9). Second, secondary bile acids (DCA/LCA) promote the activation of hepatic stellate cells via the TGR5 receptor. Activated hepatic stellate cells transform into myofibroblasts, which secrete large amounts of extracellular matrix (ECM) components, such as collagen, leading to liver fibrosis and thereby driving the progression of NAFLD (10). However, it is not clear whether the changes of gut microbiota and gut metabolites affect the occurrence and development of NAFLD through serum metabolites.

Due to the complexity and heterogeneity of the pathogenesis, involving genetic, metabolic, environmental and other factors, the treatment of NAFLD faces many bottlenecks. NAFLD therapy based on gut-liver axis and targeting gut microbiota has become a hot spot, and various new metabolic regulation drugs are under clinical development. However, how gut microbiota and its metabolites affect the development of NAFLD through serum metabolites is still unclear. Therefore, in order to study whether the changes of serum metabolites in NAFLD mice are affected by gut microbiota and gut metabolites, so as to promote NAFLD. We conducted non-targeted serum metabolome to analyze the serum metabolites of NAFLD mice, and analyzed the correlation analysis on the data of gut microbiota, gut metabolites and serum metabolites in previous experiments, in order to study their characteristics and relationship, and explore the regulatory mechanism of gut microbiota and its metabolites affecting the development of NAFLD through serum metabolites, so as to provide new targets for clinical treatment.

## 2 Methods

### 2.1 Animal feeding and sample collection

Six-week-old male C57BL/6 mice (weighing 17-19g) were purchased from Beijing Weitong Lihua Experimental Animal Technology Co., LTD and raised in a special pathogen-free (SPF) facility in the Experimental Animal Center of Southwest Medical University at an ambient temperature of 22 ± 2°C, a relative humidity of 50%-60% and a light/dark cycle of 12 hours. After a week’s adaptation period, 20 mice were randomly divided into two groups, fed for 12 weeks: CK group (n=10, Standard chow diet) and NAFLD group (n=10, High fat diet, HFD). Standard chow diet and HFD were purchased from Beijing Keao Xieli Co., LTD. See (**Supplementary Tables S2**, **S3**) for energy supply and high-fat feed formula for mice. All animals were free to drink water and eat freely. Observed the physical activity, food and water consumption and defecation of experimental mice every day. At the end of the specified feeding period, all mice were fasted overnight and anesthetized by intraperitoneal injection of 1% pentobarbital sodium (50 mg/kg body weight). Serum was collected for non-targeted metabolome detection. The data of serum metabolites are related to obesity index (Body weight), liver function index (ALT, AST), serum biochemical index (TG, TC, HDL and LDL), gut microbiome and gut metabolites, which have been presented in previous studies (11).

### 2.2 Non-targeted serum metabolome

Took samples from -80°C, slowly dissolved them at 4°C, then took 100ul samples from each group, and added 400ul precooled methanol acetonitrile solution (1:1, v/v) to each group. After vortex mixing for 60s, the samples were placed at -20°C for 1h, and then centrifuged at 14000rcf at 4°C for 20min. The obtained supernatant were collected and analyzed. Took out equal volumes of each group of processed samples and mix them to prepare quality control (QC) samples to evaluate the stability of the system during the whole experiment. After the samples pretreatment were completed, the sample were analyzed by LC-MS/MS. The pretreated samples were separated by UHPLC (Agilent 1290 Infinity LC) and combined with HILIC column. The chromatographic column temperature was 25°C and the flow rate was 0.3 mL/min. The mobile phase consists of A (water +25 mM ammonium acetate +25 mM ammonia water) and B (acetonitrile). The gradient elution procedure was as follows: 0-0.5 min, 95% B; 0.5-7min, 95%-65% B; 7–8 min, 65% to 40% B; 8–9 min, 40%B; 9-9.1 min, 40% to 95% B; 9.1–12 min, 95%B. During the whole analysis, the samples were placed in an automatic sampler at 4°C. In order to avoid the influence caused by the fluctuation of instrument detection signals, the samples were continuously analyzed in random order. QC samples were inserted into the sample queue to monitor and evaluate the stability of the system and the reliability of experimental data. The samples were separated by UHPLC and analyzed by mass spectrometer (Agilent 6550) with electrospray ionization source (ESI) for positive and negative ion modes. ESI source conditions are as follows: Gas Tem: 250°C, Drying gas: 16 L/min, Nebulizer: 20 psig, Sheath gas Tem: 400°C, sheath Gas Flow: 12 L/min, Vcap: 3000 V, Nozzle voltage: 0 V, Fragment: 175 V, Mass Range: 50-1200, Acquisition rate: 4 Hz, cycle time: 250ms. After the samples were detected, the metabolites were identified by mass spectrometer (AB Triple TOF 6600), and the primary and secondary spectrograms of QC samples were collected. ESI source conditions were as follows: Ion Source Gas 1 (Gas 1): 40, Ion Source Gas 2 (Gas 2): 80, Curtain gas (CUR): 30, source temperature: 650°C, IonSapary Voltage Floating (ISVF) ± 5000 V (positive and negative modes); The secondary mass spectrum was obtained by information dependent acquisition (IDA), and the high sensitivity mode was adopted, with de-clustering potential (DP): ± 60 V (positive and negative modes) and Collision Energy: 35 ± 15 eV. IDA was set as follows: exclude isotopes within 4 Da, candidates to monitor per cycle: 10. Data acquisition was segmented according to the mass range, 50-300, 290-600, 590-900, 890-1200, thus expanding the acquisition rate of secondary spectrogram, and each method collected four repetitions in each segment. The collected data were identified by self-built metabolite data-dependent acquisition (MetDDA) and lipid data-dependent acquisition (LipDDA) methods respectively.

### 2.3 Data processing and statistical analysis

The original data was converted into mzXML format by ProteoWizard, and then peak alignment, retention time correction and peak area extraction were carried out by XCMS program. Metabolites were identified by accurate mass number matching (< 25 ppm) and secondary spectrum matching, and the database built by the laboratory was searched. After Pareto-scaling pretreatment, the data was analyzed by multidimensional statistics, including unsupervised principal component analysis (PCA), supervised partial least squares discriminant analysis (PLS-DA) and orthogonal partial least squares discriminant analysis (OPLS-DA). Principal component analysis was used to determine the trend of intra-group polymerization and inter-group separation, while PLS-DA and OPLS-DA were used to further determine the differences between groups. OPLS-DA model passed 200 iterations of cross-validation and replacement test, and was verified based on the model’s Y change (R2Y) and predictive ability (Q2). When 1≥R2Y and Q2≥0.4, the model was determined to be stable and reliable. One-dimensional statistical analysis included Student’s t-test and variance multiple analysis to determine the significantly different metabolites between different groups. Drawing volcano map with Rstudio.

### 2.4 Differential metabolites and KEGG pathway analysis

According to the variable importance for the projection (VIP) and *P* value obtained from OPLS-DA model, the metabolites with significant differences were screened. VIP>1 and *P*<0.05 were the screening criteria. Metabolites were compared with the online Kyoto Encyclopedia of Genes and Genomes (KEGG) database (http://geneontology.org/) to retrieve their KEGG homologues (KOs) and then mapped them to the pathways in KEGG. The enrichment analysis of KEGG pathway based on Fisher exact test was applied, and all metabolites of each pathway were considered as the background data set. Only paths with *P*<0.05 were considered significant.

### 2.5 Correlation analysis

Spearman statistical method was used to analyze the correlation coefficients among serum metabolites, phenotype index, gut metabolites, gut microbiota screened in experimental samples. Combined with R(V2.15.3, http://www.R-project.org) and Cytoscape software (V3.8.2, https://cytoscape.org/), matrix heat map, hierarchical clustering and correlation network analysis were performed. This allows us to explore the relationship between serum metabolites and phenotype index, gut metabolites and gut microbiota from many angles.

### 2.6 MetOrigin

MetOrigin (http://metorigin.met-bioinformatics.cn/) was used for tracing the origin of differential metabolites. Origin analysis, functional analysis, and Sankey network analysis were all conducted using the simple MetOrigin analysis mode available on the official website.

## 3 Results

### 3.1 Analysis of serum metabolites in NAFLD mice

#### 3.1.1 The spectrum of serum metabolites has changed

In order to explore the relationship between the changes of serum metabolites and the occurrence of NAFLD, we used non-targeted metabolome to detect mouse serum. Firstly, we compared the total ion chromatography (TIC) of six QC samples in positive or negative ion mode, including retention time (RT), peak value, intensity and resolution. The TIC overlap of QC samples was good, which showed that this method was robust, highly repeatable and stable. The TIC results of the sample showed that the peak shape was complete and the adjacent peaks were well separated, which indicated that the chromatographic and mass spectrometry conditions were suitable for sample identification (**Supplementary Figures S1A, B**). Pearson correlation analysis was performed on QC samples. The correlation coefficients of the three QC samples in positive and negative ion modes were all greater than 0.99, indicating that the correlation between QC samples was good (**Supplementary Figures S1C, D**). QC samples fluctuated within the range of positive and negative standard deviation of MCC, which showed that the test data was reliable (**Supplementary Figures S1E, F**).

PCA score chart showed that the explanatory rates of normal mice and NAFLD mice in positive and negative ion modes (R2X) were R2X=0.527 and 0.579, respectively. The two groups of samples were well separated, and the same group of samples had good aggregation and repeatability (**Supplementary Figures S2A, B**). OPLS-DA supervision model was used to highlight the differences of samples between groups. In the positive ion mode of OPLS-DA, R2X=0.434, R2Y=0.994, Q2 = 0.885, while in the negative ion mode, R2X=0.462, R2Y=0.975, Q2 = 0.919 (**Supplementary Figures S2C, D**). The values of R2Y and Q2 were close to 1, which showed that the model was stable and reliable. Q2 value was about 1, which showed that OPLS-DA model had good predictability. Based on the model’s explanation of Y(R2Y) variables and the model’s prediction ability, the OPLS-DA model was verified by 7 cycles of interactive verification and 200 permutation tests. The value of Q2 intercept was less than 0, which indicated that the model was robust and reliable without fitting (**Supplementary Figures S2E, F**).

#### 3.1.2 Analysis of serum differential metabolites and KEGG pathway

We used Fold Change Analysis (FC) and Students t-test to get FC value and *P* value respectively to draw volcano map. In this experiment, the volcano map was obtained under the screening conditions of FC>1.5 and *P*<0.05, and all metabolites in positive and negative ion modes were normally distributed. Combined with the VIP value of OPLS-DA model, VIP>1 and *P*<0.05 were taken as the criteria for screening significantly different metabolites (**Supplementary Figures S3A, B**). We identified 84 significantly different metabolites, 34 in positive ion mode and 50 in negative ion mode, mainly including 43 lipids, 15 amino acids and their derivatives, 5 nucleotides and their derivatives, 4 energy metabolites, 2 vitamins, 2 bile acids and 13 other metabolites (**Supplementary Table S1**). Among them, the significant changes of 9 kinds of differential metabolites may be related to NAFLD, including Polyunsaturated Fatty Acids (Eicosapentaenoic Acid, Docosahexaenoic Acid, Linoleic Acid, Arachidonic Acid, Alpha-Linolenic Acid), Monounsaturated Fatty Acid (Eicosenoic Acid) and Branched-Chain Amino Acids (L-Threonine, L-Valine, L-Leucine).

**Figure 1:**
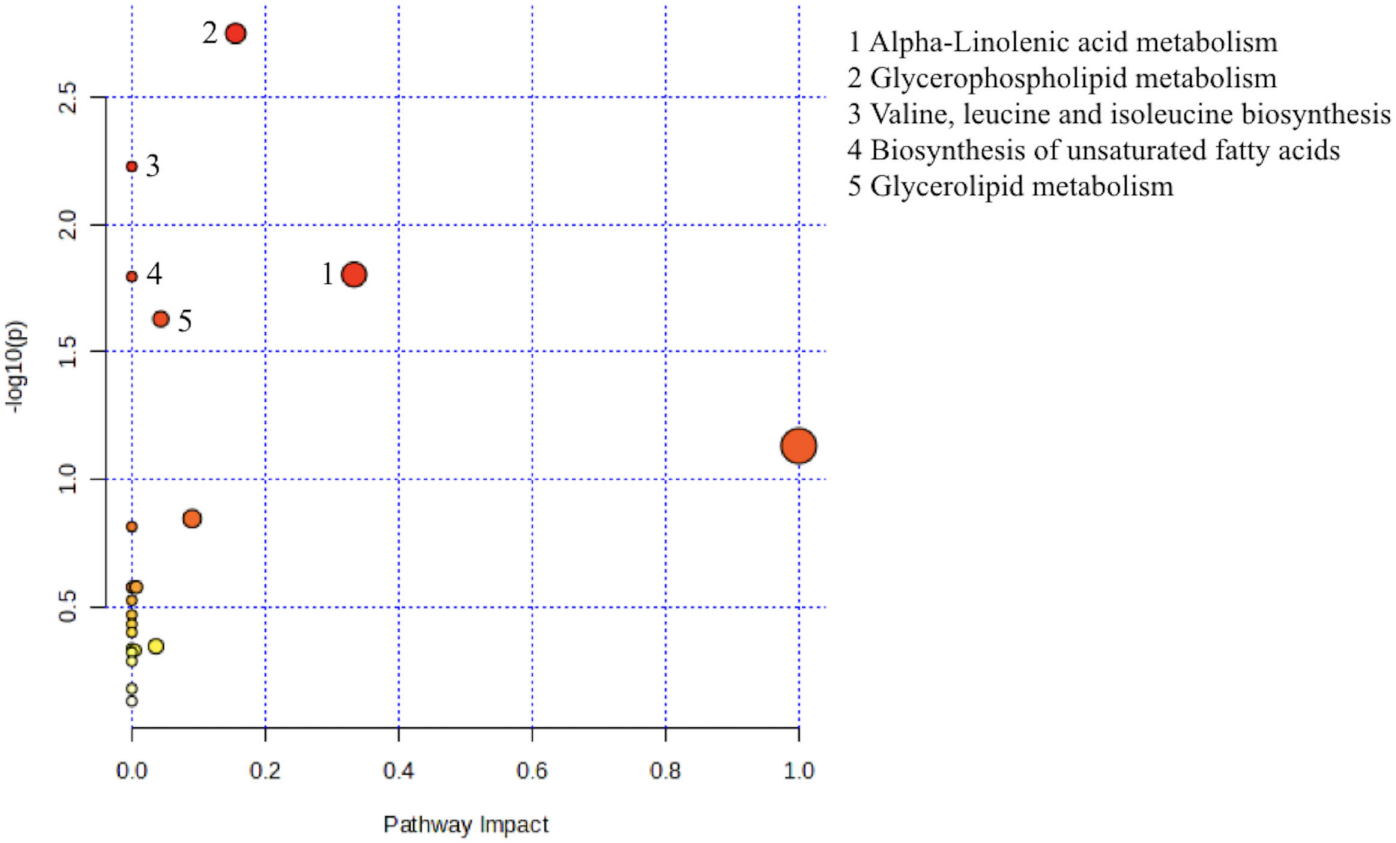
Metabolic pathway analysis using MetaboAnalyst 6.0 (http://www.metaboanalyst.ca). x-axis, pathway impact; y-axis, −log (P). Circles represent metabolic pathways. Darker circles indicate more significant changes in the metabolites in the corresponding pathway, whereas the size of the circle corresponds to the pathway impact score.

Subsequently, we looked up the differential metabolites in KEGG database. In order to further determine the biological significance of differential metabolites, we used MetaboAnalyst software to analyze metabolic pathways. According to the results of enrichment analysis, the *P* value of Glycerophospholipid metabolism, Valine, Leucine and isoleucine biosynthesis, Alpha-Linolenic acid metabolism, Biosynthesis of unpredictable fatty acids, Glycerolipid metabolism were statistically significant (*P*<0.05, as shown in the Y axis) (**Figure 1**).

### 3.2 Correlation analysis

#### 3.2.1 Correlation analysis between serum metabolites and phenotype index

**Figure 2:**
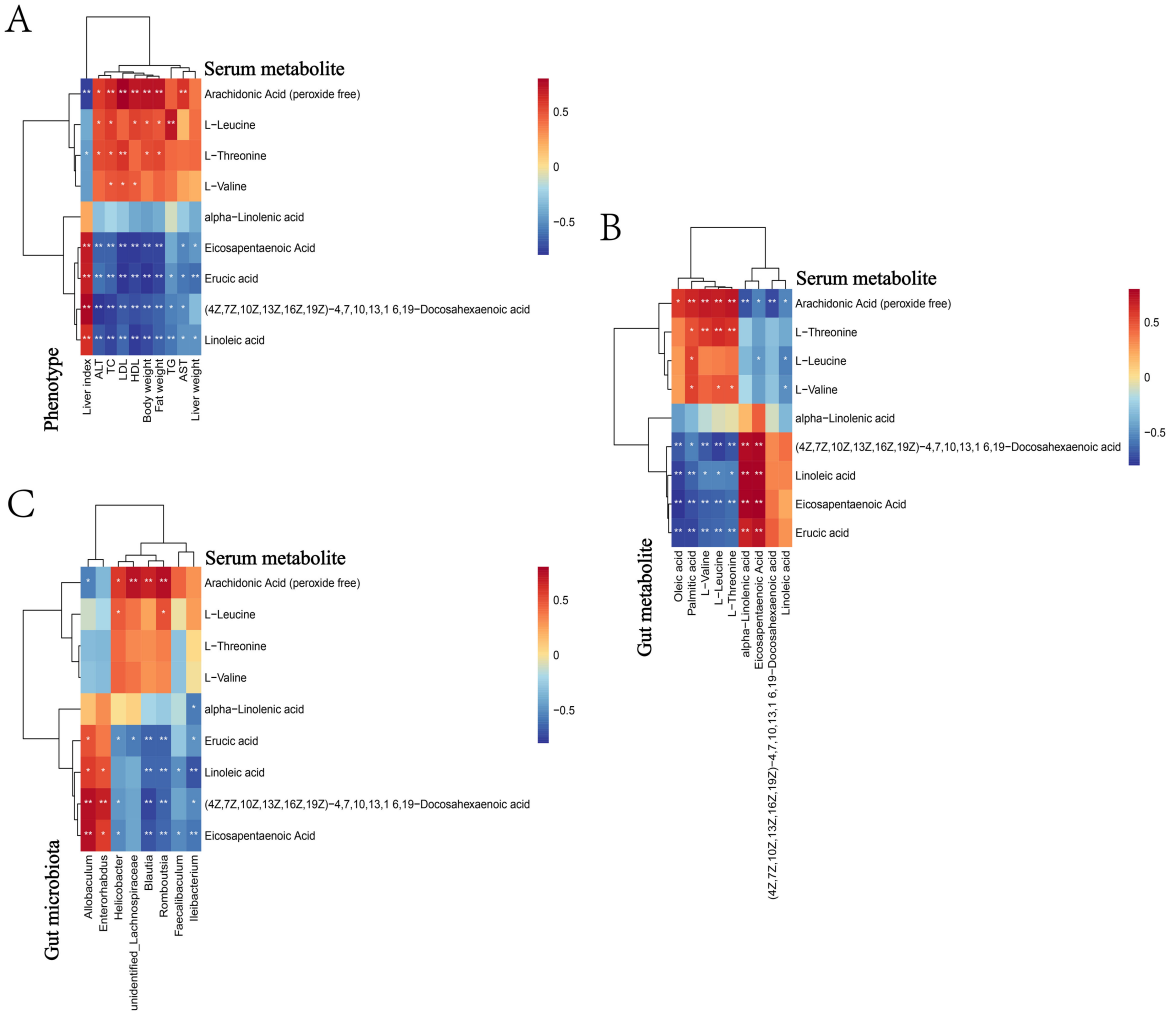
Heat map for correlation analysis between serum metabolites and phenotype, gut metabolite and gut microbiota. Heat map for phenotype **(A)**, gut metabolite **(B)** and gut microbiota **(C)**. The correlation coeffcient r is shown in color. r>0 represents a positive correlation and is shown in red; r<0 represents a negative correlation and is shown in blue. The darker the color, the stronger the correlation. “*” indicates P<0.05, and “**” indicates P<0.01.

Through metabonomics analysis, we screened out 84 serum metabolites with significant differences in NAFLD mice, and further correlation analysis revealed the relationship between these metabolites and blood lipid, liver function and obesity index (**Figure 2A**). There were 34 serum metabolites positively correlated with liver function (ALT, AST), blood lipids (TG, TC, HDL, LDL) and obesity index (lipid weight, body weight, liver weight), and negatively correlated with liver index, which meat that the levels of these metabolites may increase with the increase of obesity and disease risk. Among them, Arachidonic Acid (AA), L−Leucine, L−Threonine, L−Valine, etc., these metabolites were elevated in the intestine of NAFLD mice and found to be significantly elevated in the serum. There were 29 kinds of serum metabolites which were contrary to the above correlation, indicating that the levels of these metabolites may decrease with the decrease of obesity degree and disease risk, including: EPA, DHA, Linoleic acid (LA), Alpha−Linolenic acid (ALA), Erucic acid (EA), etc., these metabolites decreased in the intestine of NAFLD mice and were found to be significantly reduced in serum. These serum metabolites may play an important role in the pathogenesis of NAFLD.

#### 3.2.2 Correlation analysis of serum metabolites and gut metabolites

We analyzed the correlation between serum and gut differential metabolites (**Figure 2B**), and the results showed that, 9 serum metabolites, namely AA, EPA, DHA, LA, ALA, EA, L-Threonine, L-Leucine and L-Valine, were found to be related to gut metabolites. Serum AA was negatively correlated with gut LA, EPA, DHA and ALA, while serum EPA and DHA were positively correlated with gut EPA and DHA. In addition, serum LA, ALA and EA were positively correlated with gut EPA and DHA, and serum L-Threonine, L-Leucine and L-Valine were positively correlated with gut L-Threonine, L-Leucine and L-Valine. These results suggested that specific metabolites in serum may participate in the development of NAFLD by affecting gut metabolites, but the specific mechanism was still unclear.

### 3.3 Source of serum metabolites and host-microbiota function analysis

**Figure 3:**
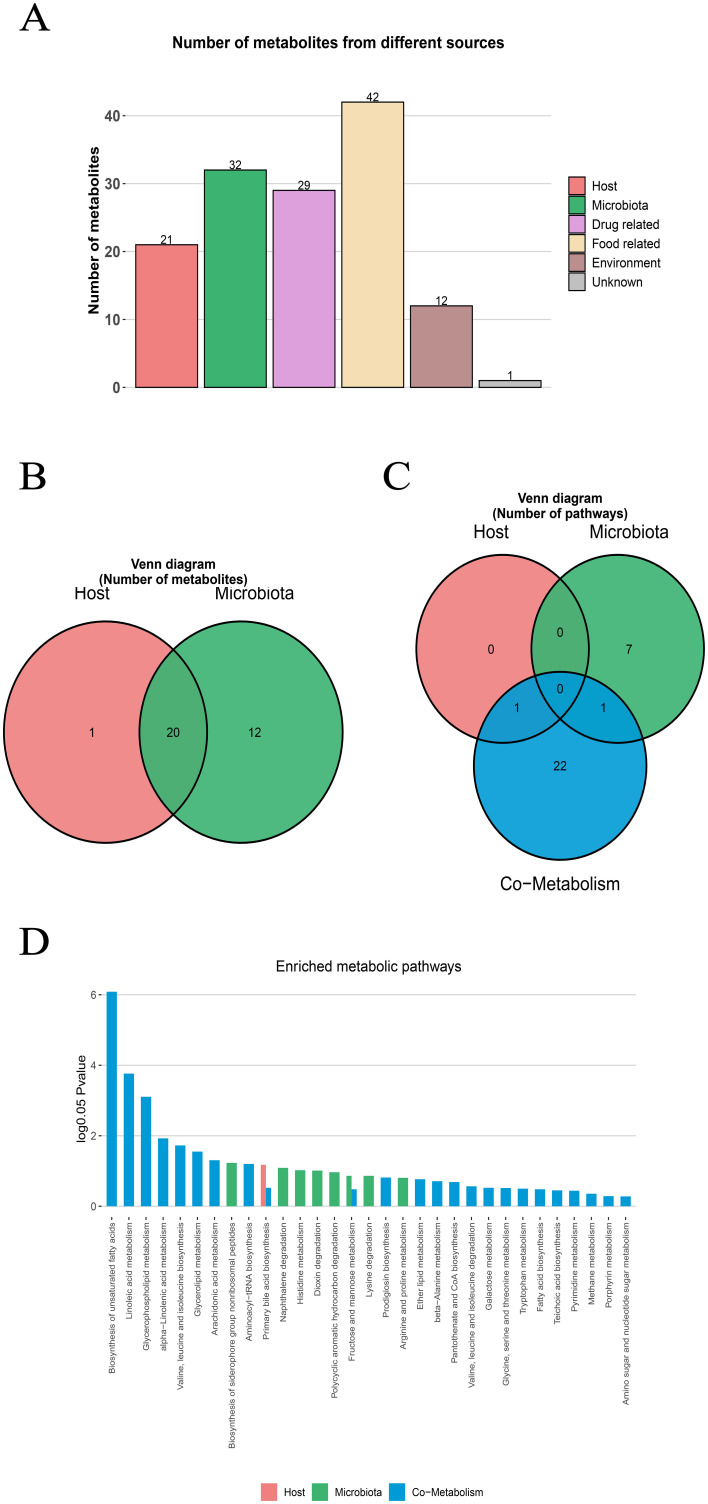
MetOrigin tracing analysis of differential metabolites. **(A)** Bar plot of the number of metabolites in different sources **(B)** Venn diagram of the number of metabolites in the human and bacterial communities. **(C)** Venn diagram of the number of metabolic pathways in the human and bacterial communities. **(D)** Bar plot of enriched metabolic pathways in the human and bacterial communities.

The metabolite-microbiota origin analysis of MetOrigin showed that the metabolites in NAFLD mice serum came from many sources. The analysis identified 20 microbiota-host co-metabolites, 1 host-specific metabolite and 12 microbiota specific metabolites. In addition, the metabolites related to food (42 species), drugs (29 species), environment (12 species) and unknown sources (1 species) were identified (**Figures 3A, B**). Functional analysis showed that 1, 8 and 24 metabolic pathways respectively corresponded to host, microbiota and co-metabolism data (**Figure 3C**). Among the important metabolic pathways related to NAFLD (log0.05 *P*>1), Lipid metabolisms accounted for the largest proportion, and mainly involved host-microbial co-metabolism (**Figure 3D**). The three co-metabolic pathways, Biosynthesis of unsystematic fatty acids, Linolenic acid metabolism and Glycerophospholipid metabolism, had the greatest influence (log0.05 *P*>2), and their combined effects led to metabolic disorder.

In addition, Bio-Sankey network was used to explore the statistical correlation and biological relationship between microbiota and metabolites (**Supplementary Figure S4**). Specifically, in the Biosynthesis of unsaturated fatty acids, this pathway involved 5 different metabolites (namely LA, ALA, EPA, DHA and AA) which participated in 5 different metabolic reactions (R08177, R08178, R08179, R08180, R08183). Through metabolomics and microbiomics analysis, the complex origin of serum metabolites in NAFLD mice model and their role in microbiota-host co-metabolism pathway were revealed.

### 3.4 Correlation analysis of serum metabolites and microbiome in NAFLD mice

The possible correlation between microbiota and identified metabolites was evaluated by Spearman rank correlation test (**Figure 2C**). For example, at the generic level, *Allobaculum* was positively correlated with serum metabolites ω-3 PUFAs (EPA, DHA, ALA) and negatively correlated with L-Threonine, L-Valine and L-Leucine. *Helicobacter* and *Blautia* were just the opposite to the above, which were negatively correlated with serum metabolites EPA, DHA and ALA, and positively correlated with L-Threonine, L-Valine and L-Leucine.

### 3.5 Correlation analysis of serum metabolites and gut metabolites, gut microbiota and phenotype index

**Figure 4:**
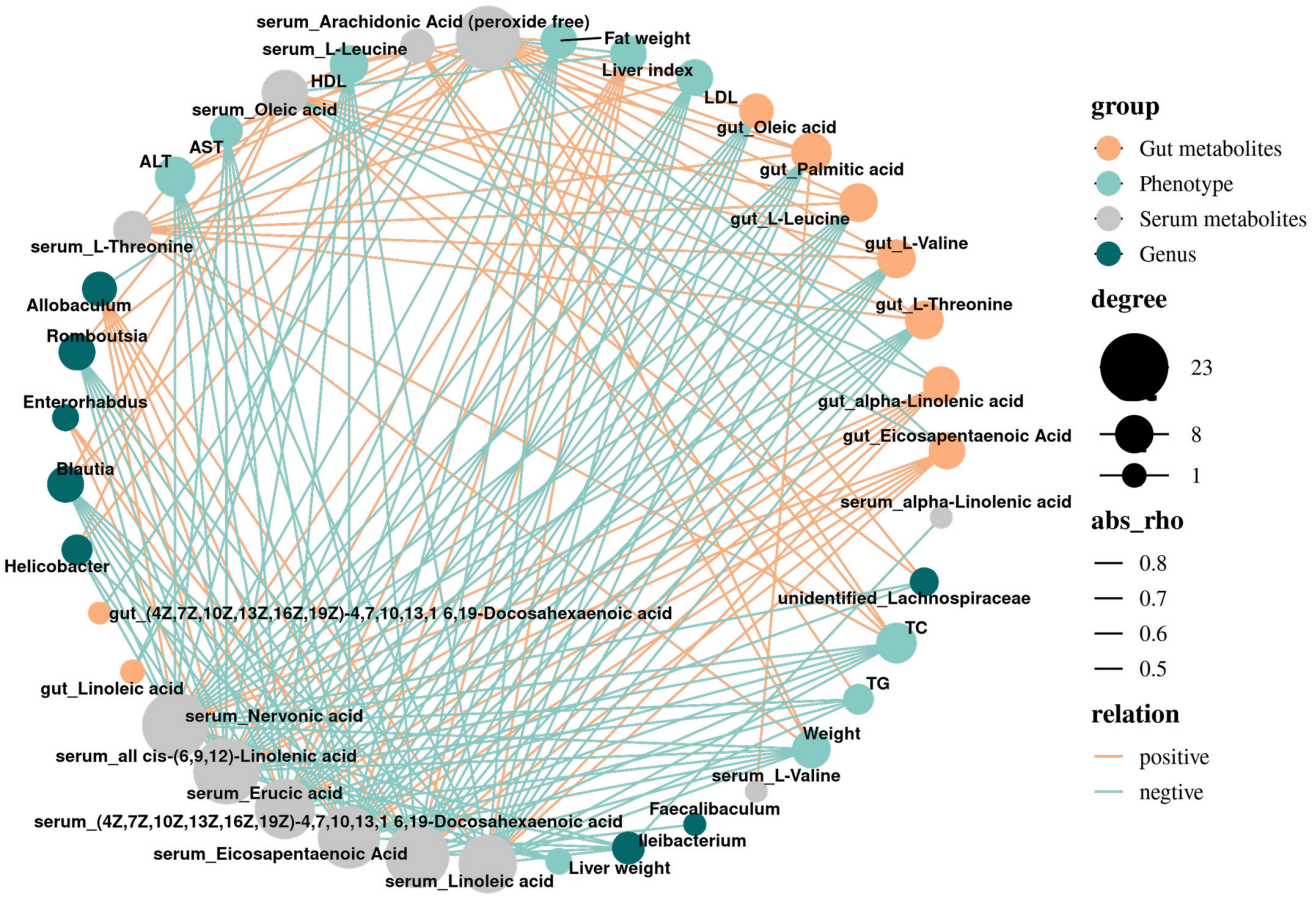
Correlation network diagram of differential metabolites, phenotype, and gut microbiota. Grey dots denote Serum metabolites; blue dots denote Phenotype; orange dots denote gut metabolites; green dots denote Genus; the size of dots indicates the quantity of correlation; and the thickness of a line represents the size of the correlation coefficient r, r>0, positive correlation, shown in orange; r<0 representatives a negative correlation, shown in blue.

In the previous study, we found that the bacterial abundance of 8 main genera in NAFLD mice changed through LEfSe analysis, and made a network diagram of these bacteria and the main changed metabolites (**Figure 4**). It was found that the abundance of *Helicobacter*, *unidentified_Lachnospiraceae*, *Blautia*, *Romboutisa*, *Faecalibaculum* and *Ileibacterium* increased significantly. Gut and serum EPA was negatively correlated with *Blautia* and *Helicobacter*, and positively correlated with *Allobaculum*. At the same time, serum L-Threonine, L-Valine and L-Leucine were negatively correlated with *Allobaculum*. The increase of *Blautia* and *Helicobacter* abundance may be beneficial to the synthesis of TG in mice and promote the accumulation of cholesterol in hepatocytes, while the decrease of *Allobaculum* abundance may lead to the increase of fat content, resulting in worse liver condition.

**Figure 5:**
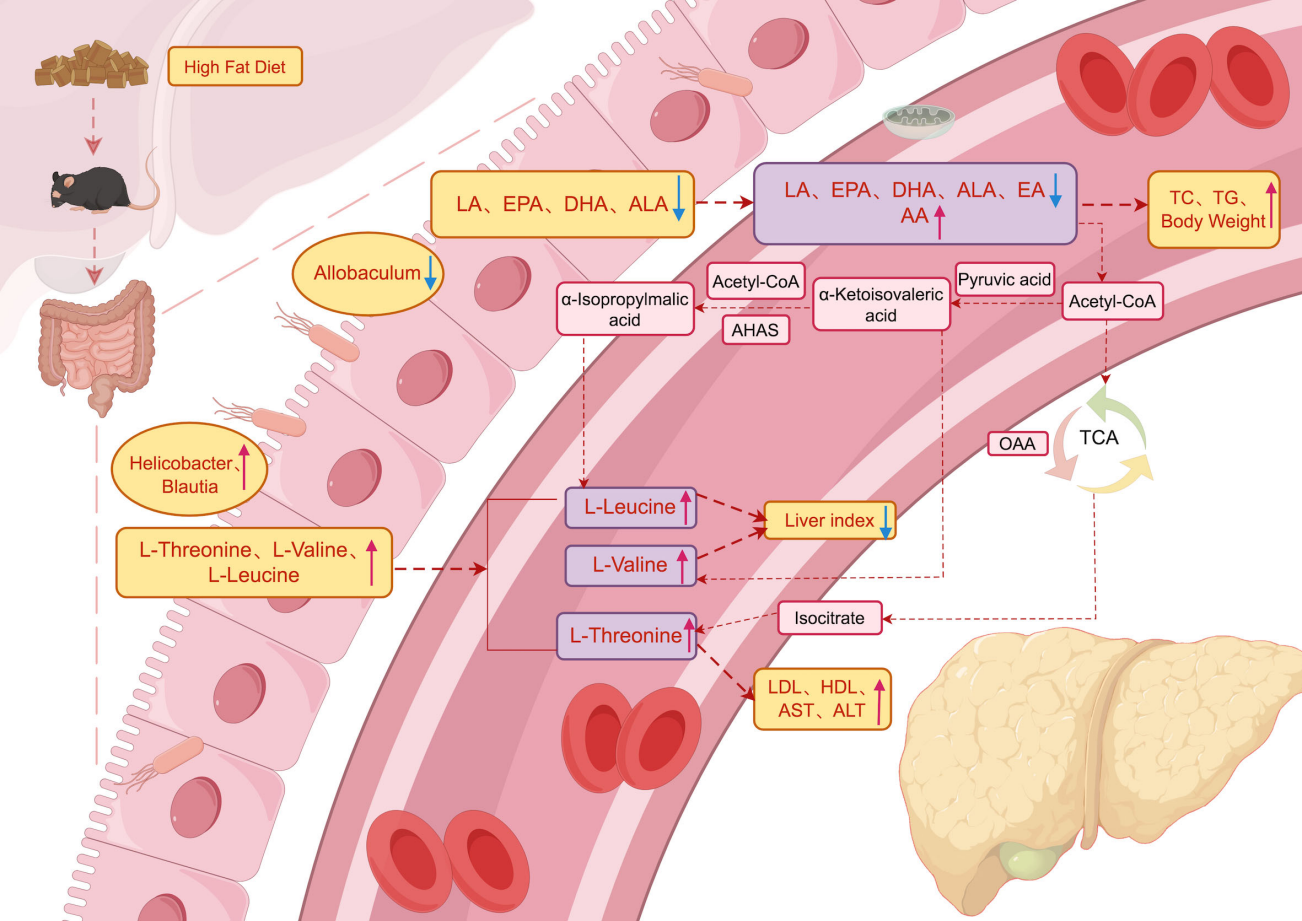
Metabolic pathways. Schematic illustration of the relationship among gut microbiota, gut metabolites and serum metabolites and its effect on NAFLD. The blue arrowhead represents the decrease, the red represents the increase, and the black font indicates that the process is supported by literatures.

In a word, we made a metabolic pathway diagram based on the interaction mechanism or potential mechanism among serum metabolites, gut metabolites and gut microbiota (**Figure 5**) (12–14). The underlying mechanism may be the imbalance of gut pathogenic microbiota *Blautia*, *Helicobacter* and beneficial microbiota *Allobaculum* in NAFLD mice, which induces gut metabolic disorder, reduces the content of unsaturated fatty acids in serum and accumulates branched fatty acids, which may aggravate the index of liver injury in mice, thus aggravating the occurrence and development of NAFLD.

## 4 Discussion

The pathogenesis of NAFLD was multifactorial and not only related to feeding behavior. Therefore, the induced NAFLD might differ from the natural pathogenesis. Nevertheless, in this study, an NAFLD animal model was successfully established through a high-fat diet. Through metabolomics analysis, nine major serum metabolites in NAFLD mice, including PUFAs, MUFAs, and BCAAs, underwent significant changes. Specifically, the levels of serum metabolites AA, L-threonine, L-valine, and L-leucine were significantly increased, while the levels of EPA, DHA, LA, ALA, and EA were significantly decreased. These changes might be related to the physiological mechanisms of serum metabolites and were of great significance in the study of NAFLD.

Serum EPA and DHA were two important ω-3 PUFAs, which had a wide range of physiological functions in the human body, including regulating blood lipids, reducing triglyceride levels in the blood, decreasing blood coagulation, lowering blood pressure, alleviating inflammation, and maintaining cardiovascular health. EPA and DHA have been shown to be useful in the synthesis of a series of novel specific lipid mediators (SPMs) (15). Studies have indicated that supplementation with ω-3 PUFAs could reduce fatty liver and significantly improve key biochemical features of NAFLD (16). EA has been proven to reduce endogenous triglyceride levels and increase the rate of triglyceride hydrolysis in the heart, indicating its potential role in maintaining lipid balance (17). A meta-analysis including 2,630 participants showed that ALA significantly reduced triglycerides, low-density lipoprotein cholesterol, and very-low-density lipoprotein cholesterol (18). LA was involved in the synthesis of phospholipids and was very important for maintaining the fluidity and function of cell membranes (19). AA was considered the true pro-inflammatory molecule among ω-6 PUFAs, generating pro-inflammatory eicosanoids such as prostaglandins, thromboxanes, and leukotrienes under the catalysis of enzymes like COX, LOX, and CYP450 (20). A study found that a high-fat diet promoted an increase in AA levels, with inflammation development, expression of inflammatory enzymes, content of lipid peroxidation products, and damage to the oxidative system observed in the first week of the experiment, indicating that changes in AA levels might be an early indicator of irreversible changes in the progression of inflammation and NAFLD (21). AA was also involved in the development of many diseases, such as hepatic fibrosis, obesity, diabetes, and colorectal cancer (22). As BCAAs, L-threonine, L-valine, and L-leucine were essential amino acids in the human body, participating in various metabolic processes in the liver. Their appropriate levels might be beneficial to the liver, while high concentrations might contribute to the development of NAFLD. In liver diseases, it has been proven that L-threonine could reduce characteristic liver damage by synthesizing phospholipids and oxidizing fatty acids and was a landmark monitoring target for monitoring liver function recovery, which might be beneficial to NAFLD (23). The intake of L-valine was negatively correlated with the risk of NAFLD, indicating that increasing L-valine intake might help reduce the risk of NAFLD (24). Additionally, L-leucine has been identified as a Sirt1 activator, acting on the AMPK-eNOS-Sirt1 pathway and capable of reversing mild NAFLD in preclinical mouse models (25). However, studies have shown that high concentrations of BCAAs, such as L-valine and L-leucine, might be associated with an increased risk of NAFLD. The increase in BCAA levels might lead to NAFLD by affecting mitochondrial function and inflammatory responses, while mitochondrial dysfunction, oxidative stress, and insulin resistance might further lead to the accumulation of BCAAs, resulting in the occurrence and development of NAFLD (26). Therefore, serum EPA and DHA could positively prevent and improve NAFLD by improving lipid metabolism and regulating inflammatory responses, reducing the risk of liver inflammation and fibrosis. EA and ALA might reduce triglyceride and cholesterol levels by regulating blood lipids. Inflammation and bile acid metabolism in serum AA might exacerbate the occurrence and development of NAFLD, while BCAAs levels might have a dual effect on NAFLD.

The changes in the nine serum metabolites associated with the NAFLD phenotype index might be influenced by gut metabolites. For example, gut metabolites such as SCFAs could reduce lipolysis in adipocytes by activating the G protein-coupled receptor GPR43, which might in turn lower the levels of free fatty acids in the blood (27). In this study, correlation analysis between serum and gut metabolites revealed that serum AA was negatively correlated with gut EPA and ALA. The conversion of ALA to EPA required desaturation and elongation reactions. These conversions of PUFAs involved competitive desaturases and elongases (28), and EPA could inhibit the conversion of AA to pro-inflammatory mediators because EPA was preferentially acted upon by these enzymes. Therefore, EPA competitively interfered with AA (29). However, in high-fat diet-induced NAFLD mice, the levels of EPA were reduced, which weakened this beneficial effect. Moreover, a significant positive correlation was found between serum EPA and gut EPA. The reduction of both gut and serum EPA in NAFLD mice indicated that the decrease in gut EPA further affected the reduction in serum EPA. This was because dietary EPA was first absorbed in the gut, and gut cells had specific transporters (such as FATP) that transported EPA from the gut lumen into the gut cells and then into the bloodstream. Therefore, the effective absorption of EPA by the gut was a prerequisite for the increase in serum EPA levels. Thus, gut metabolites EPA and ALA could regulate the levels of serum AA and EPA. The reduction of EPA and ALA weakened their inhibitory effect on AA-related inflammation, thereby affecting the formation and development of NAFLD.

The changes in the nine serum metabolites associated with the NAFLD phenotype index might be influenced by the gut microbiota. Previous studies have found that *Allobaculum* could inhibit the development of NAFLD. As a protective gut bacterium, it was negatively correlated with insulin resistance, diabetic symptoms, obesity, body weight, and inflammation. In contrast, *Helicobacter* and *Blautia* were significantly increased in NAFLD mice and were considered intestinal pathogens that promoted the development of NAFLD (11). Correlation analysis revealed that *Allobaculum* was significantly positively correlated with serum EPA. Supplementation with ω-3 PUFAs (such as EPA) was closely related to the improvement of obesity in mice and the reduction of hepatic fat accumulation, with a significant enrichment of *Allobaculum* in feces (30). Studies have shown that the abundance of *Allobaculum* was negatively correlated with serum levels of L-threonine, L-valine, and L-leucine (31), which was consistent with our findings. *Allobaculum* could inhibit the differentiation of adipocytes and fat accumulation by producing butyrate, which might affect serum BCAAs levels, as BCAAs were associated with obesity and insulin resistance (32). *Allobaculum* might also influence the valine, leucine, and isoleucine biosynthesis pathways by producing its metabolites, SCFAs (33). Correlation analysis revealed that *Helicobacter* and *Blautia* were significantly negatively correlated with serum EPA and positively correlated with serum L-threonine. ω-3 PUFAs supplementation had a protective effect against gastrointestinal diseases caused by *Helicobacter*, downregulated arachidonic acid (AA), upregulated antioxidant enzymes, and reduced lipid peroxidation. It could also inhibit the expression of inflammatory mediators in *Helicobacter*-infected cells (34). NAFLD patients exhibited BCAAs metabolic disorders, with elevated levels of BCAAs in blood and urine (35). A study by (25) has confirmed that L-threonine could reduce characteristic liver injury by synthesizing phospholipids and oxidizing fatty acids, which was beneficial for NAFLD and served as a landmark monitoring target for liver function recovery. L-leucine could also reverse mild NAFLD. All these findings indicated that the gut microbiota could influence host metabolic changes by regulating gut and serum metabolites. Therefore, *Allobaculum* was associated with a reduced risk of NAFLD and might exert its protective effects by modulating serum BCAAs and PUFAs levels, while the presence of *Helicobacter* and *Blautia* might be related to impaired gut barrier function and inflammatory responses, thereby affecting the homeostasis of serum BCAAs and PUFAs.

In addition to the influence of gut microbiota and metabolites, chronic low-grade inflammation also played a significant role in the pathogenesis and progression of NAFLD. The spleen, as a major immune organ, was involved in regulating NAFLD-related immune dysregulation through the “liver-spleen axis” (36). For example, an increase in pathogens might promote the abnormal activation of myeloid-derived suppressor cells (MDSCs) and natural killer T cells (NKT) in the spleen, which was closely related to the inflammation of fatty liver. It was worth noting that the absence of T and B cells in the liver was not reflected in the splenic lymphocyte profile. Correlation analysis has confirmed a selective strong positive correlation between the distribution of MDSCs and NKT cells in the spleen and the liver, indicating that the liver-spleen axis regulated obesity-induced immune dysregulation in a cell-specific manner (37). Dysbiosis of the gut microbiota affected the host’s metabolic and immune status through the gut-liver axis and might further exacerbate the pathological progression of NAFLD through the “liver-spleen axis,” especially by influencing the distribution of immune cells in the spleen and liver.

The potential mechanism may be the imbalance between the pathogenic microbiota Blautia and Helicobacter and the beneficial microbiota Allobaculum in the gut of NAFLD mice, which induces gut metabolic disorders, leading to a decrease in serum PUFAs (EPA, DHA, LA, ALA, and EA) and an accumulation of BCAAs (L-threonine, L-valine, and L-leucine) and AA. This in turn exacerbates hepatic injury markers in mice, thereby aggravating the occurrence and development of NAFLD, with EPA and DHA playing particularly important roles. In summary, the changes in serum metabolites indicate that EPA and DHA may be potential targets for improving gut microbiota disorders and NAFLD in mice, while the levels of BCAAs may have a dual effect on NAFLD. However, further research is needed to elucidate the underlying mechanisms.

## 5 Conclusions

The main serum metabolites of NAFLD mice have changed significantly, among which BCAAs (L-Threonine, L-Valine, L-Leucine) and AA are up-regulated, while PUFAs (EPA, DHA, LA, ALA) and MUFA (EA) are down-regulated, which may further aggravate the formation of fatty liver, thus aggravating the development of NAFLD. Gut pathogenic microbiota *Blautia*, *Helicobacter* and beneficial microbiota *Allobaculum* regulate the levels of serum unsaturated fatty acids and BCAAs, especially EPA and DHA, which leads to a significant increase in liver injury index, which may aggravate the development of NAFLD and obesity in mice. The level of BCAAs may have a dual effect on NAFLD, and moderate intake may help reduce the risk of NAFLD, while high concentration may lead to the development of NAFLD. In a word, regulating serum metabolites through gut microbiota and its gut metabolites may provide a new perspective and potential target for the prevention and treatment of NAFLD.

## Data Availability

The datasets presented in this study can be found in online repositories. The names of the repository/repositories and accession number(s) can be found below: https://www.ebi.ac.uk/metabolights/, MTBLS12668.
