# Supplementary material for: Gut microbiota regulates serum metabolites in mice with nonalcoholic fatty liver disease via gut metabolites: mechanisms involving branched-chain amino acids and unsaturated fatty acids
Source: Front Endocrinol (Lausanne). 2025 Jul 22;16:1606669. doi: 10.3389/fendo.2025.1606669 (PMC12321561; doi:10.3389/fendo.2025.1606669)
Supplement: Supplementary file 1 [file DataSheet1.zip › Supplementary material/Supplementary figure.docx]

Supplementary Material

**
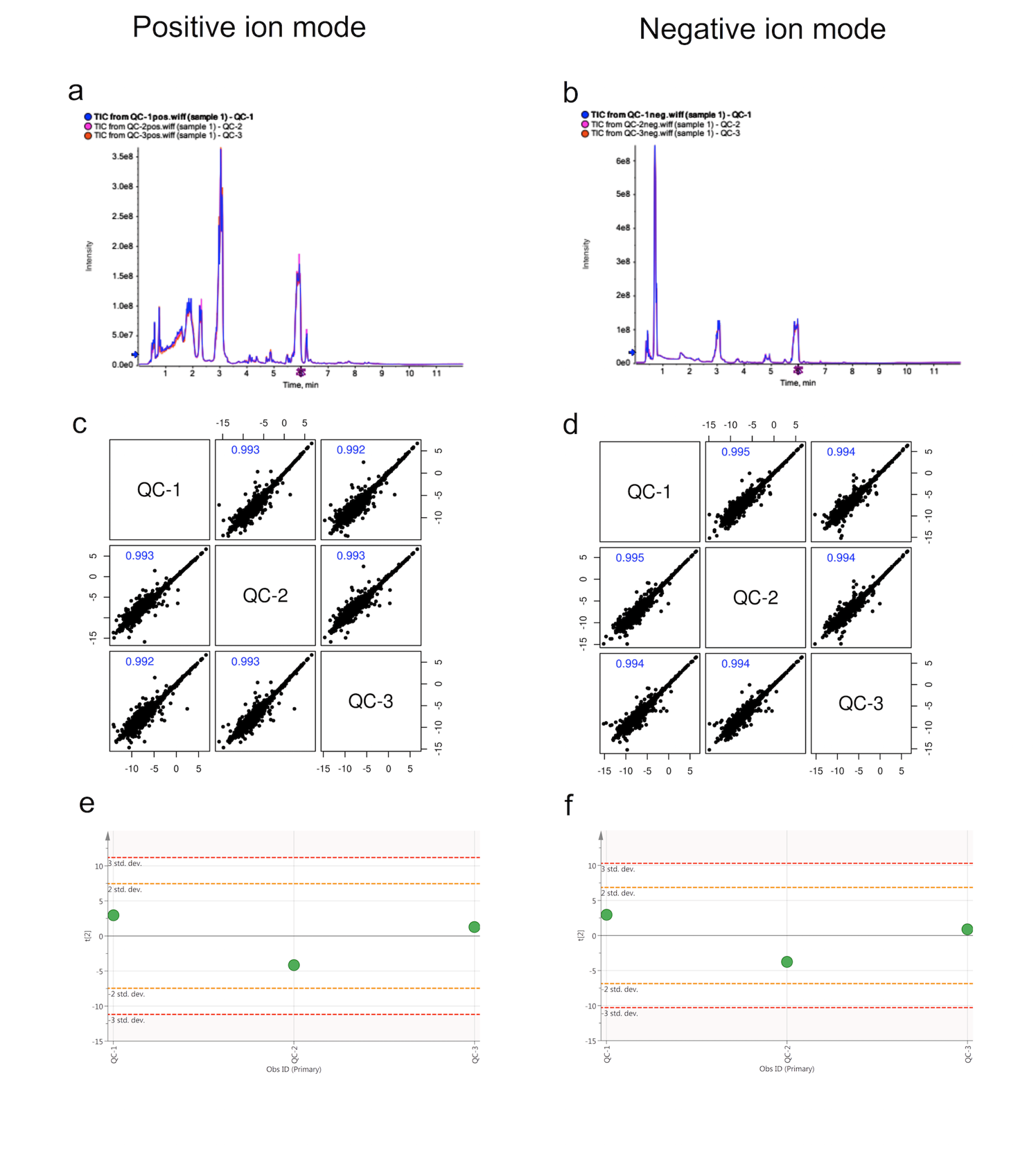
**

**Fig.S1. Reliability evaluation of cecal contents metabolomics in NAFLD and Normal mice**. **(a-b)** Total ion chromatogram of QC sample; **(c-d)** Correlation map of QC samples; **(e-f)** Multivariable control chart (MCC) of QC sample. **(a, c, e)** in positive ion mode, **(b, d, f)** in negative ion mode.


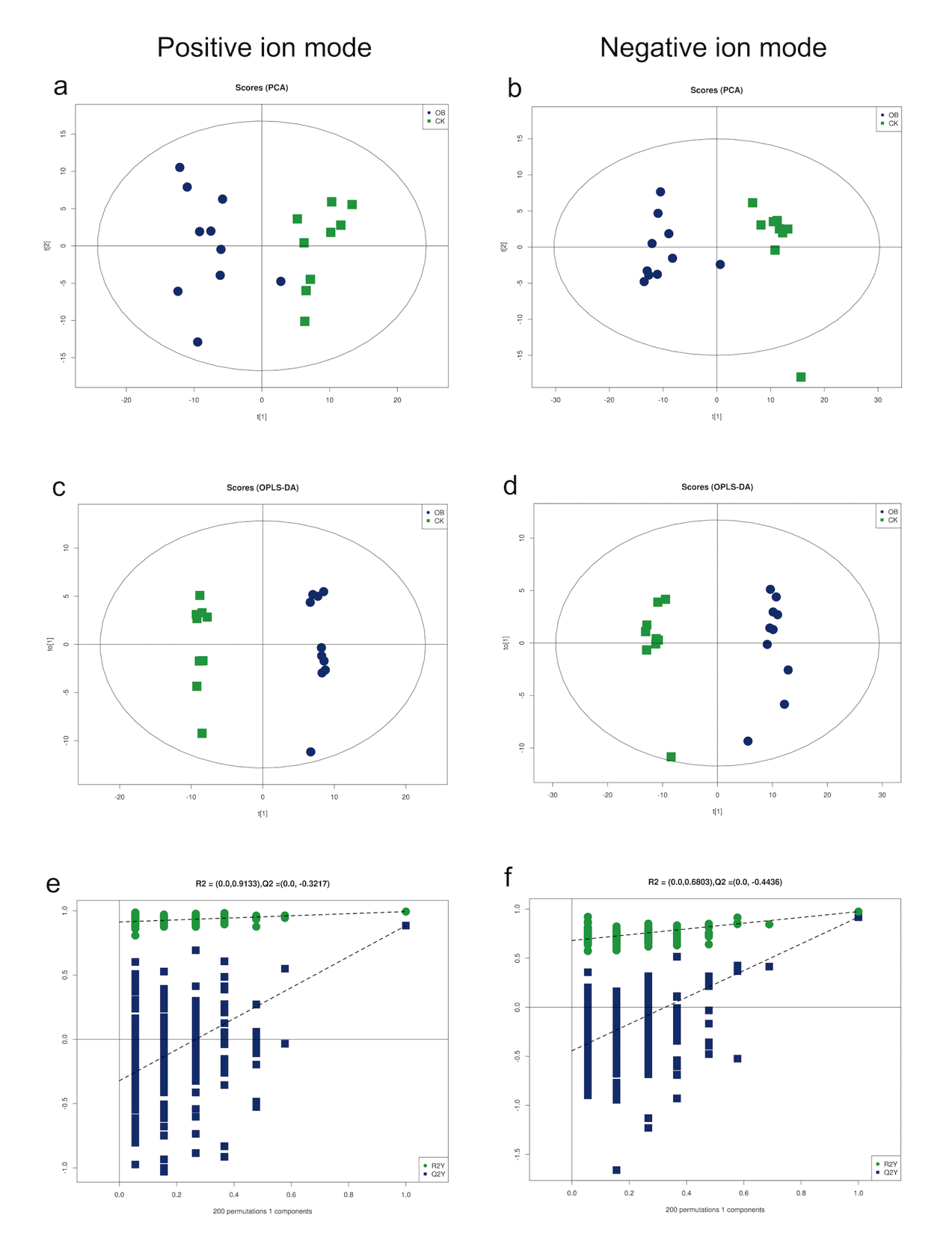


**Fig.S2.** **Non-targeted metabolomics performed with LC-MS/MS reveals microbial and host related metabolome changes** **in NAFLD and Normal mice**. **(a-b)** Principal component analysis (PCA) score plot, t[1] = first principal component, t[2] = second principal component. **(c-d)** Orthogonal partial least square discriminant analysis (OPLS-DA) of scores, t[1] = first principal component. t[2] = second principal component. **(e-f)** OPLS-DA score plot in the positive and negative ion mode. (e-f) permutation test plot of OPLS-DA. **(a, c, e)** in positive and ion mode, **(b, d, f)** in negative ion mode.

**
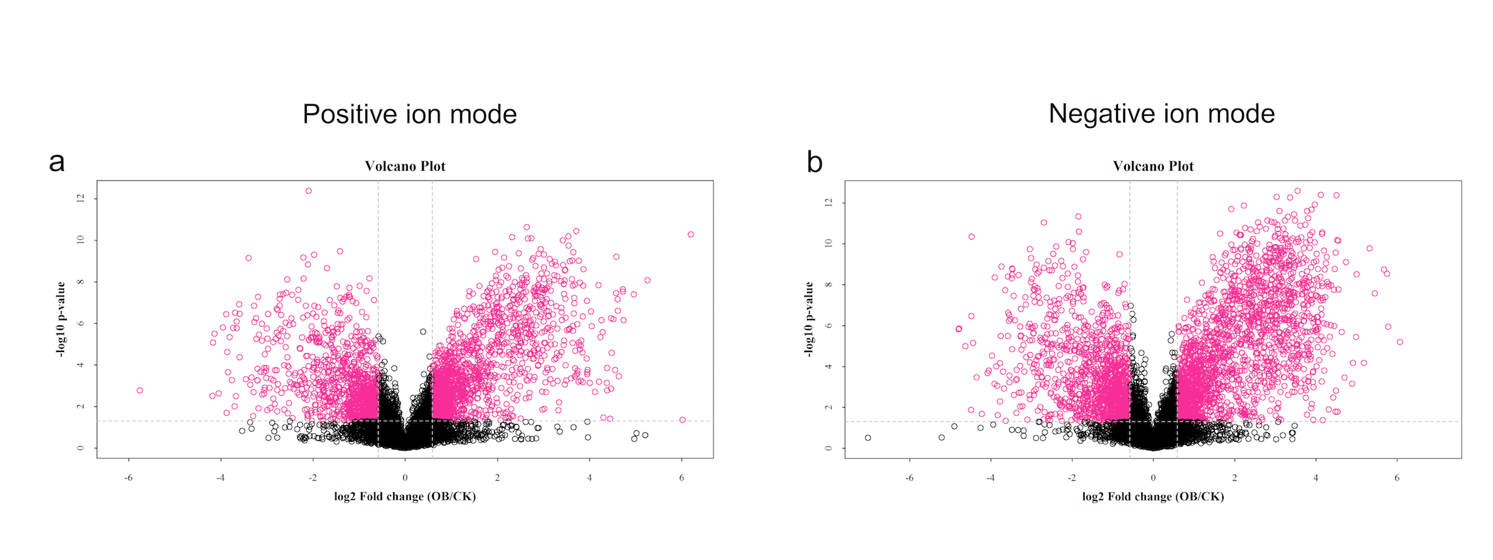
**

**Fig.S3. Volcano plots presenting the differential metabolites (red) between Control and NAFLD mice**. **(a)** in positive and ion mode, **(b)** in negative ion mode. Fold Change (FC) >1.5, *P*-value <0.05 (Student’s *t*-test)


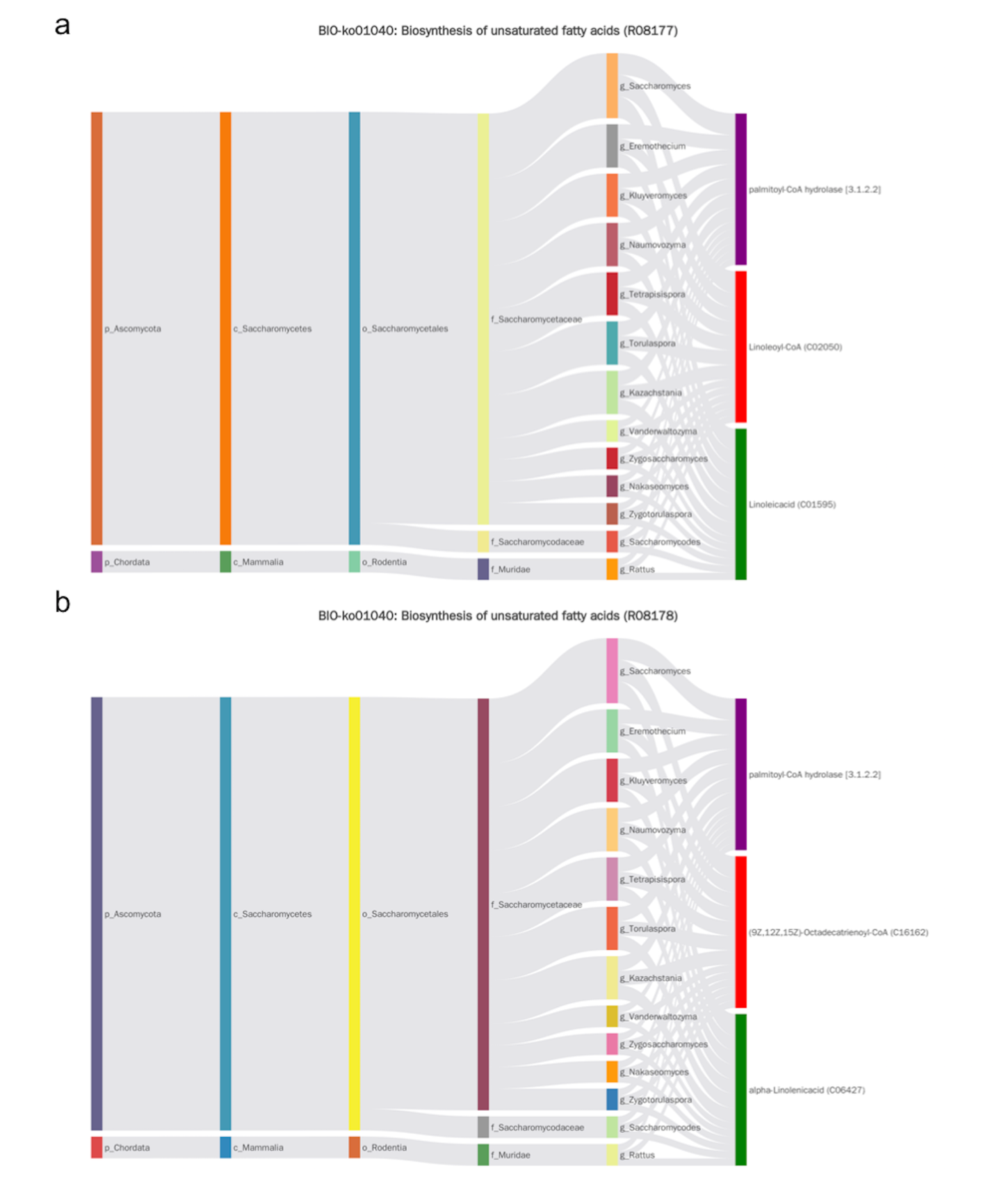


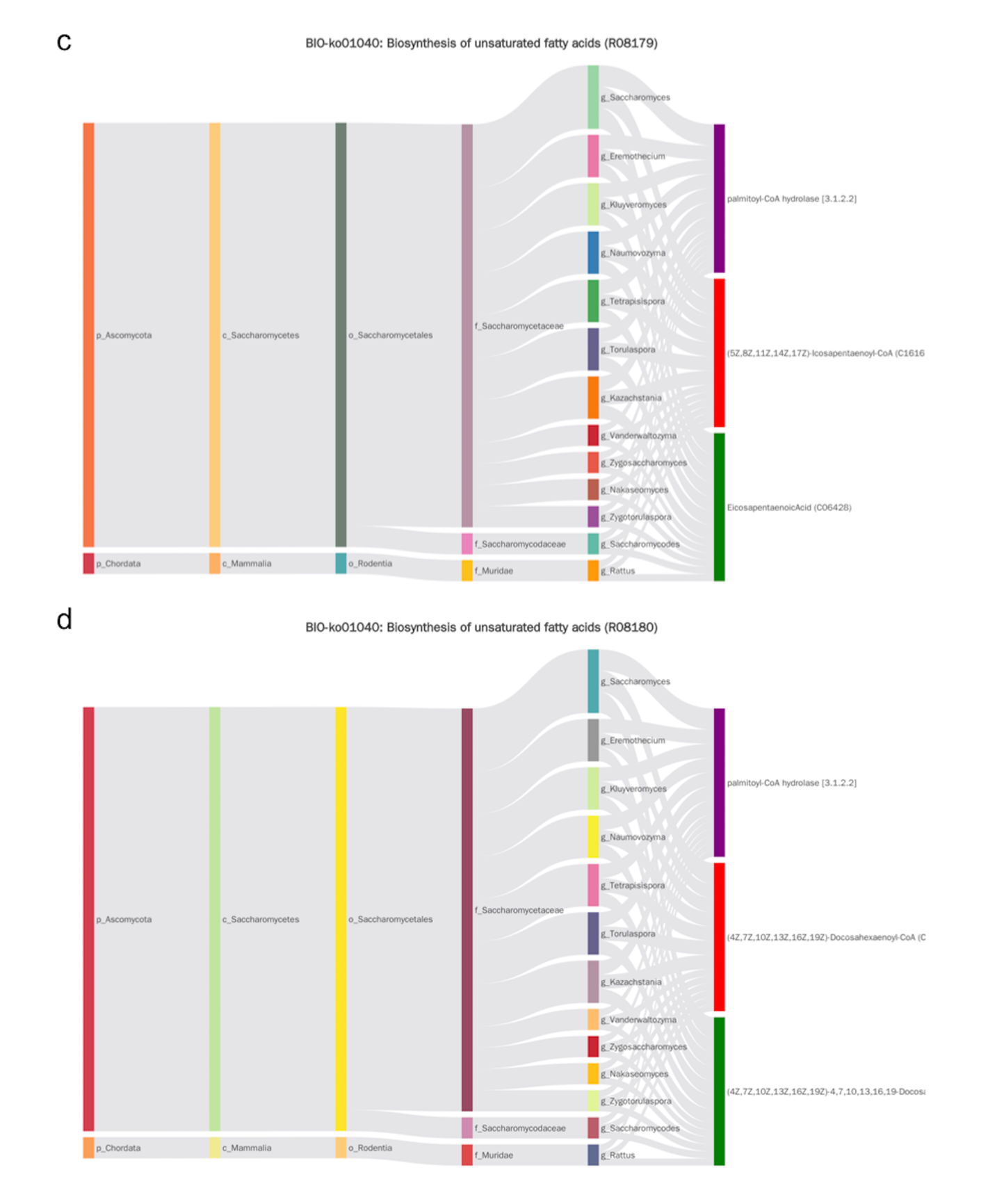


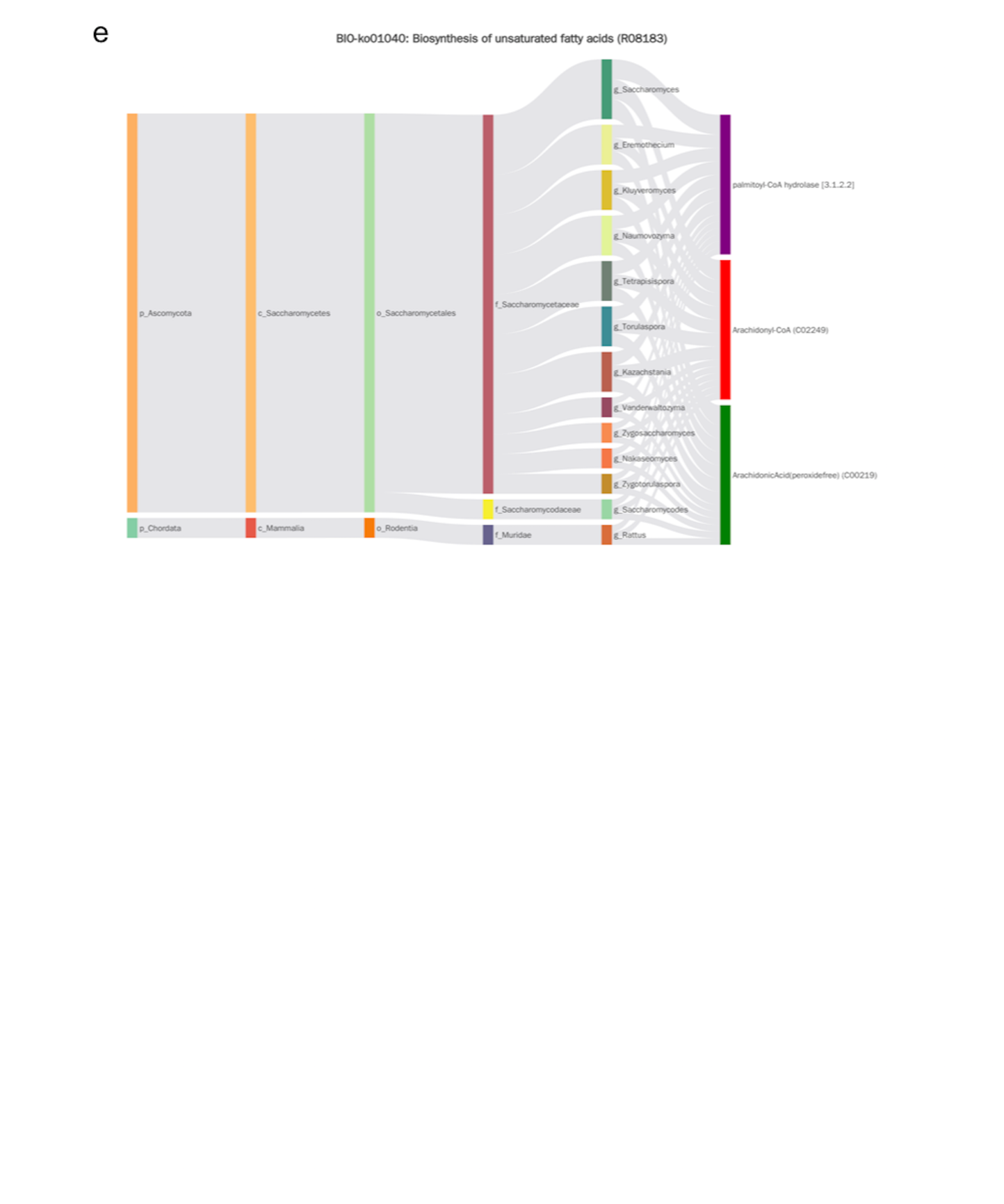


**Fig.S4. Sankey network of MetOrigin analysis.**
